# Aerated Waters

**Published:** 1905-01-28

**Authors:** 


### Aerated Waters.

The twelfth report of the Medical Officer for the County of London, to some of the general features of which we have already referred, contains some truly amazing statistics concerning the quantity of so-called "aerated waters" consumed in England, and chiefly, it is said, in the metropolis and in the north. The facts, as far as they can be ascertained, are set forth in a special report by Dr. Hamer on the manufacture of these compounds; and the figures dealt with, like those which express astronomical distances, are of such a magnitude as to baffle the imagination. On the authority of Mr. Idris, Dr. Hamer declares that 70,000 persons and 22,000 horses are employed in the industry in the United Kingdom, that the capital invested is between 30 and 40 millions of pounds, and that the total consumption is over 300 million dozen half pints. Dr. Hamer assumes, from various data set forth more or less fully in his report, that, on an average, every man, woman, and child in the kingdom consumes a quarter of a pint of aerated water daily; and the question why they should do so becomes more puzzling the more it is considered. It is recorded of an American that he condemned abstinence from alcoholic drinks on the ground that, in Holy Writ, he "found uncommon small mention of water as a beverage," and that the only person who desired to have any was that unfortunate Dives whose fate he desired to shun. It does, nevertheless, seem inconceivable that, with the supplies of the new Water Board freely available, so large a number of persons should seek to contaminate what poets describe as "the pure element," not only with carbonic acid gas, but also with a great variety of physicky abominations of different kinds. Dr. Hamer attributes the custom, in some degree, to the fact that our potable waters, by the filtrations and other processes necessary in order to present them to the consumer in a state of satisfactory purity, are deprived of a freshness which is grateful to the palate, and that this is in a certain degree restored by the artificial aeration. To some extent, no doubt, this may be so, but the explanation is hardly adequate to the magnitude of the phenomena to be explained. It is apparent, not only from the quantities of these waters consumed, but also from the extent to which they are manufactured of a low price in poor neighbourhoods, often by foreigners of highly objectionable type, and for the supply of localities containing almost exclusively a poor population, that they are regarded in some degree as a necessary, and that there must be an ignorant prejudice against mere water, as such, sufficiently powerful to induce an enormous expenditure upon substitutes of pence and halfpence which would be far better devoted to the purchase of nourishing food. There is an old story of a famous wine-taster who made a bet that, when blindfolded, he would pronounce correctly upon the nature of anything which was given him to drink. He confessed himself beaten by a glass of water. He felt sure that he had never tasted that particular liquid before, but he affirmed without hesitation that it was "very nice." The late Mr. Justice Maule, too, is reported to have given a famous decision, in the case of a locked-up jury, that water was not "drink" within the meaning of the Act; but, in spite of the judge and of the expert, we would strongly urge upon the consumers of some of the messes described by Dr. Hamer that they should at least give water a trial. We cannot conceive that anyone in possession of his senses, who had once tasted pure water, should wish it to be disguised by any of the common methods, or should waste halfpennies in compassing the adulteration of a fluid which may be had of good quality for nothing.

